# Editorial: Social and Administrative Policy in Healthcare and Pharmacy Practice

**DOI:** 10.3389/fpubh.2022.901847

**Published:** 2022-05-10

**Authors:** Kingston Rajiah, Shazia Qasim Jamshed, Mohamed Izham Mohamed Ibrahim

**Affiliations:** ^1^Gandhi Institute of Technology and Management (GITAM) School of Pharmacy, GITAM University Hyderabad, Hyderabad, India; ^2^Faculty of Pharmacy, Sultan Zainal Abidin University, Kuala Terengganu, Malaysia; ^3^College of Pharmacy, QU Health, Qatar University, Doha, Qatar

**Keywords:** medication adherence, outcome evaluation (health care), disease management, medication use, patient behavior

Most public health policies and activities in developing and developed countries are government-funded, so new information should be open to the public (1). There is a need to focus on both strengths and weaknesses of medication use policy, medication marketing, and evaluation of theoretical models. Furthermore, these could impact practice and/or patient behavior in responses to the social, health, and environmental challenges providing both theoretical and empirical findings. Potential issues include but are not limited to medication products/programs/services, medication adherence, disease management, medication use policy, and medication marketing (2–4). Social and health issues related to delivering health care services, medical governance, medication management, and pharmaceutical management related to multilevel, multi-stakeholder, and multi-sectoral approaches to healthy and affected communities should be explored. It is noteworthy to criticize ethical issues related to medication products/programs/services, medication use policy, and medication marketing. The ideas relevant to the social policy and health policy-related concepts received contributions from health policymakers, academics, practitioners, and collaborators in other sectors whose work impacts social and administrative policy. They were the appropriate sources to discuss how policy and practice change over time, how it compares across the globe, and how it is realized at all levels, from international to local. Whilst focused on relevance to practice, it was understood that examining the theories and philosophies that underpin social and administrative policy was essential. It captured a diversity of opinions across a broad range of fields, from the traditional (medication adherence; disease management; medication use policy; medication marketing, etc.) to the new (big data, new technologies). This Research Topic provided a venue for health professionals in social and administrative policy disciplines with a specific interest in policy and practice to share their research findings and other Research Topics related to public health.

The publications under this Research Topic highlighted key components like quality use of medicines, drug utilization, pharmacy care services, Potentially Inappropriate Medication (PIM), pharmacoeconomics, and pharmaceutical policies.

A qualitative exploration of the medication-taking behavior among Indian immigrant diabetics in Australia highlighted spontaneity in initiating their treatment as prescribed. Still few postponed starting their treatment due to preconceived notions about side effects and adverse effects related to medication, while other few resorted to Ayurveda forms of treatment. Those who did not receive expected results from an alternative form of treatment switched to a conventional modality. Long-term discontinuation was also reported. Akram et al. executed this research in a way that the “in-depth information” extracted participants' experiences and perspectives with contemplative explications.

The systematic review cum meta-analysis by Bhagavathula et al. is a vivid account of the regional variations of the prevalence of both polypharmacy, hyper polypharmacy, and inappropriate medication use in different Indian states. The authors skillfully undertook the concept of risk of bias in all 27 included studies which were then followed by performing meta-analyses. We cannot deny a myriad of cultures and practices in different geographic regions, which in turn, are the points of limitations in the current systematic review cum meta-analyses. The conclusive remarks reported extensively high polypharmacy and hyper polypharmacy coupled with traces of inappropriate medication use.

Another systematic review by Xu et al. highlighted the issue of inappropriate medicine use for stress ulcer prophylaxis (SUP) in intensive care patients and how this has been handled by clinical pharmacists' interventions. The authors carefully tackled study selection and quality assessment of included studies but deferred meta-analyses due to the heterogeneity of participants. Interestingly, the review reported that despite clinical pharmacists' interventions the extent of inappropriate use of SUP pharmacotherapy during ICU transfer is high, and recommended to include the pharmacists' suggestions for discontinuation of SUP pharmacotherapy. Although no cost-effectiveness analysis was performed a few studies in the review reported that the intervention from clinical pharmacists generated economic benefits in strengthening and improving SUP pharmacotherapy.

The research from UAE researchers on substandard and falsified medicines and understanding of and identification of counterfeit medicines among the lay public deserve merit to be discussed. Although a descriptive cross-sectional study but as it was executed in different regions of UAE through a web-based validated tool, El-Dahiyat et al. reported a sparse understanding of the identification of counterfeit medicines and recommended the need for educational campaigns to sensitize the lay public about the efficacy and safety of medicines but also to emphasize the importance of avoiding counterfeits.

Pharmacists generally provide patient care services either solo or sometimes conjointly as active members of the patient care team and generate consolidated health outcomes and improved satisfaction rates followed by a substantial decrement in healthcare costs. One cannot deny the significant role of pharmacists as immunizers, public health specialists, organizers of health and wellness screening programs, and medication therapy managers in recent years. Few of the published articles in this Research Topic account for how pharmacists can be involved in weight management programs and exercised potential influence on diabetes and hypertension.

In a Malaysian study, Verma et al. extensively explored how community pharmacists contributed to weight management and the challenges and facilitators involved in their role extension. Fulfilling the basic criteria of executing qualitative research, they reported that community pharmacists are instrumental in exercising influence in weight management programs and expressed their readiness in imparting educational advice and lifestyle modifications coupled with medication and supplement counseling and referrals to other healthcare professionals. Also reported challenges like paucity of time and proper space allocation along with reimbursement issues, they advocated following substantial remuneration models for community pharmacists involved in weight management schemes.

Likewise in a randomized, controlled, single-blinded, pre-post intervention study from Pakistan, community pharmacists are reported to be involved in diabetes and hypertension care. Malik et al. assessed the effects of pharmacist counseling on blood pressure and glucose control among patients attending community pharmacies and reported better knowledge in diabetes and hypertension in patients enrolled in the intervention group.

Another research from South Africa within the paradigm of pharmacy care services generated a rewarding vision of the predicaments in the line of clinical pharmacy practice and envisaged the recent profile of clinical pharmacists and their roles and responsibilities. It was reported that when working in hospital wards they performed many functions which include both clinical and logistical but recommended to have certification system need to be in place which will standardize the practice of clinical pharmacy services in different facilities.

Timely research from China advocated setting up and divulge a tele pharmacy support system to execute pharmaceutical care during the COVID-19 pandemic. Under the aegis of the Beijing Pharmacists Association, a remote pharmacy service model was set up to provide medication consultation services using WeChat App. The constructed “Cloud Pharmacy Care” platform had attracted more than 1,400 viewers and 66 followers within 2 months followed by more than 35 cases of patient counseling. The forte of this interactive consultation model strengthened the medication therapy management aspect for chronically ill patients and reported superior compliance and dissemination of safe medication knowledge.

Another research from United Arab Emirates UAE promptly recommended the role of clinical pharmacists in combating readmissions and rehospitalizations in heart failure patients and explicated non-compliance with medications as the major cause of rehospitalization.

A unique study from researchers in Bulgaria also tapped the importance of adherence to medications in acromegaly and advocated instituting a national level guideline that not only has methods of assessment but also going to deal with the improvement of adherence in acromegaly patients. In this stepwise study where first the literature review was done along with an analysis of Bulgarian legislative documents, the researcher Kamusheva et al. did a pilot study for the assessment of the level of treatment adherence among hospitalized patients followed by the development of the plan for the implementation of specific guidelines BULMEDARCO Bulgarian Guideline For Medication Adherence Assessment And Improvement In Acromegaly.

The economic evaluation provided evidence for the quality of health care delivery improvement and health outcomes. The uptake of evidence-based practices assures the efficient use of limited healthcare resources. Four studies investigated the economic aspect of medicines. A study by Cai et al. measured the cost-effectiveness of camrelizumab in treating patients with advanced or metastatic esophageal squamous cell carcinoma. It is evident that camrelizumab as a second-line therapy is cost-effective in terms of its QALY compared to chemotherapy. Economic burden and financing sources of off-label oncology treatment were assessed by Gordon et al. They discovered that the main sources of funding were private health insurance. In addition, the average monthly cost of off-label treatment was 4–5 times much higher than the net average household monthly income. Lee et al. studied the trends of pharmaceutical expenditures using Korean National Health Insurance claim data. They indicated that the increase in the number of drugs used as the driver of the increase in prescription drug spending. Li H. et al. evaluated the price effect of the volume-price contract initiative on pharmaceutical supplies to public hospitals in China. They found that the reduction of the unit price of procured cardiovascular medicines is associated with the volume-price contract initiative. The initiative worked well for cardiovascular medicines, but the impact varied for other medicines.

Potentially Inappropriate Medication (PIM) use are linked with numerous adverse effects and mortality in Geriatrics (5). In this Research Topic, a study by Bhagavathula et al. demonstrated that the prevalence of PIM use among the geriatric population is high in India. Oncology medicines give rise to many challenges for policymakers while the pricing of products due to increasing uncertainty during marketing authorization with variation in combination regimens, cost-effectiveness and budget impact. In this Research Topic, a study by Cai et al. reported that the oesophagal squamous cell carcinoma patients' quality of life could improve with camrelizumab, be cost-effective, and reduce adverse reactions. Another study by Chen et al. stated that increased price and decreased affordability as barriers to access anticancer essential medicines. Another study by Liu quoted that public medical insurance is an essential means of preventing uncertainty and avoiding health risks among the people who cannot access health. A study by Gordon et al. stated that in a comprehensive healthcare system, the financing sources of the off-label treatments may influence access to it. In most countries, expenditures on healthcare have increased after implementing the national health insurance system covering medications (6). In this Research Topic, a study by Lee et al. revealed that increased prescription drug spending was mainly due to an increase in the number of drugs used. Another study by Li Z. et al. stated that the volume-price contract initiative has the potential to bring down the price of pharmaceutical supplies. According to WHO, counterfeit pharmaceutical products are fraudulently and deliberately mislabeled similar to the source (7). A study on this Research Topic by El-Dahiyat et al. mentioned that drug counterfeiting is a menace to any nation's economy and public health. Hence, awareness among the public is essential.

## Author Contributions

All authors listed have made a substantial, direct, and intellectual contribution to the work and approved it for publication.

## Conflict of Interest

The authors declare that the research was conducted in the absence of any commercial or financial relationships that could be construed as a potential conflict of interest.

## Publisher's Note

All claims expressed in this article are solely those of the authors and do not necessarily represent those of their affiliated organizations, or those of the publisher, the editors and the reviewers. Any product that may be evaluated in this article, or claim that may be made by its manufacturer, is not guaranteed or endorsed by the publisher.
